# Phylogenetic Relationships of the Fern *Cyrtomium falcatum* (Dryopteridaceae) from Dokdo Island Based on Chloroplast Genome Sequencing

**DOI:** 10.3390/genes7120115

**Published:** 2016-12-02

**Authors:** Gurusamy Raman, Kyoung Su Choi, SeonJoo Park

**Affiliations:** Department of Life Sciences, Yeungnam University, Gyeongsan 38541, Korea; bioramg@gmail.com (G.R.); kschoi01@ynu.ac.kr (K.S.C.)

**Keywords:** *Cyrtomium falcatum*, holly fern, chloroplast genome, polypod ferns, molecular clock

## Abstract

*Cyrtomium falcatum* is a popular ornamental fern cultivated worldwide. Native to the Korean Peninsula, Japan, and Dokdo Island in the Sea of Japan, it is the only fern present on Dokdo Island. We isolated and characterized the chloroplast (cp) genome of *C. falcatum*, and compared it with those of closely related species. The genes *trnV-GAC* and *trnV-GAU* were found to be present within the cp genome of *C. falcatum*, whereas *trnP-GGG* and *rpl21* were lacking. Moreover, cp genomes of *Cyrtomium devexiscapulae* and *Adiantum capillus-veneris* lack *trnP-GGG* and *rpl21*, suggesting these are not conserved among angiosperm cp genomes. The deletion of *trnR-UCG*, *trnR-CCG*, and *trnSeC* in the cp genomes of *C. falcatum* and other eupolypod ferns indicates these genes are restricted to tree ferns, non-core leptosporangiates, and basal ferns. The *C. falcatum* cp genome also encoded *ndhF* and *rps7*, with GUG start codons that were only conserved in polypod ferns, and it shares two significant inversions with other ferns, including a minor inversion of the *trnD-GUC* region and an approximate 3 kb inversion of the *trnG-trnT* region. Phylogenetic analyses showed that *Equisetum* was found to be a sister clade to Psilotales-Ophioglossales with a 100% bootstrap (BS) value. The sister relationship between Pteridaceae and eupolypods was also strongly supported by a 100% BS, but Bayesian molecular clock analyses suggested that *C. falcatum* diversified in the mid-Paleogene period (45.15 ± 4.93 million years ago) and might have moved from Eurasia to Dokdo Island.

## 1. Introduction

The genome of the plant organelle chloroplast (cp) is responsible for photosynthesis [1,2] and provides rich evolutionary and phylogenetic information [3,4]. These genomes are also involved in the primary functions of the plant such as starch storage; sugar synthesis; production of several amino acids, lipids, vitamins, and pigments; and important sulfur and nitrogen metabolic pathways [5]. Most fern plant cp genomes contain 117–124 unique genes within a 131–168 kilobase (kb) circular chromosome, with genome size differences mostly due to variations in the length of inverted repeats (IRs) and small single copy (SSC) regions [6]. When compared to angiosperm cp, many fern cp genomes display significant rearrangements, including a 30 kb inversion, followed by a 3.3 kb inversion and a D inversion [7]. The key mechanism of gene order change is inversion due to intramolecular recombination, which primarily occurs through dispersed repeats of the cp genome [8,9]. However, evolutionary hot spots representing significant levels of insertions and deletions (indels) with high frequencies of base substitutions are concentrated in specific genes and intergenic spacers [9]. Although gene number and order are comparatively conserved in the cp genome of ferns, there are some differences among taxa [10]. Fern plants hold a critical phylogenetic position as the extant sister group to seed plants [11]; therefore, understanding the organization and evolution of fern plastomes provides useful information for comparative studies across land plants [12].

Islands have long been significant systems in ecology and evolutionary biology, and they present several unique characteristics that are useful for biological studies [13]. Dokdo Island consists of two main islets, Seodo (western island) and Dongdo (eastern island), as well as 89 smaller surrounding islets. Dokdo Island is situated between the Korean Peninsula and Japan at distances of 215 km and 250 km, respectively, and was formed by an underwater volcanic eruption during the late Pliocene epoch. Recent research suggests that the volcanic edifice of Dokdo Island formed first (sometime between 4.6 and 2.5 million years ago (mya)), after which its underlying tectonic plate may have shifted, facilitating the formation of the volcanic body of the nearby Ulleungdo Island [14] approximately 2.7–0.01 mya [15,16]. Despite similar origins, Dokdo Island is different from other nearby islands and the mainland in terms of its information, features, fauna, and ecosystem. It is also the oldest island in the Korean Peninsula. Because it is an oceanic island that is disconnected from the mainland and other islands, it is critical to study its history, island biogeography and plant evolution [17,18]. Several studies of its flora, vegetation, and ecology have been conducted [19]; however, no studies have investigated the evolution of plants on Dokdo Island using complete chloroplast genomes. The genus *Cyrtomium* is a small member of the *Dryopteridaceae* family, which consists of 42 species and is sister to the *Polypodium* ferns that formed a clade with the Eupolypods [10]. *Cyrtomium falcatum* is a species of house holly or Japanese holly fern that originated in Asia, but is currently cultivated in several countries as an ornamental plant. It is an evergreen perennial fern endemic to forested areas or shady cliffs on Dokdo Island, the Korean Peninsula, and Japan. It is, however, the only fern plant found on Dokdo Island. Interestingly, the rhizome of this plant is used medicinally as an anthelmintic, chiefly for the expulsion of tapeworms [20]. Because its unique attributes, we are very interested in studying the molecular phylogenetic and evolutionary relationships of this particular fern species. In this study, we report the complete cp genome sequence of *C. falcatum*. In addition to describing the structure of the cp genome, we also provide comparative analyses of the cp genome sequences of closely related species of ferns. We also present the results of phylogenetic analyses of DNA sequences for 76 protein-coding genes from *C. falcatum* and 23 other fern cp genomes. We also conducted molecular evolutionary analyses to elucidate the origin of this fern plant. Overall, this comparative genomics study will contribute to an increased understanding of phylogenetic relationships among ferns and the evolution of *C. falcatum* on Dokdo Island.

## 2. Materials and Methods

### 2.1. DNA Sequencing

All genomic DNA was extracted from fresh, young leaves of the *C. falcatum* plant by using a modified cetyl trimethylammonium bromide method [21]. The plant material from Dokdo island is triploid and apogamous. High quality DNA was sequenced using the Illumina NextSeq 500 sequencing system (LabGenomics, Seongnam, Korea). The paired-end library was constructed with an insert size of 550 base pairs (bp). Sequence trimming, assembly, and mapping were performed using CLC Genomics Workbench, v7.0.4 software (CLC-Bio, Aarhus, Denmark). Chloroplast genome reads were aligned to the closest cpDNA sequence obtained from *Cyrtomium devexiscapulae* (GenBank accession number: KT599100) [10]. The depth of coverage for the cp genome of *C. falcatum* was 256X. Consensus sequences were extracted and gaps were filled by polymerase chain reaction (PCR) amplification using specific primers based on the gap between sequences. PCR products were purified and sequenced using conventional Sanger sequencing. Sequencing data and gene annotation were then submitted to GenBank and assigned accession number KP189363.

### 2.2. Genome Analysis of the *C. falcatum* Chloroplast Genome

Initial annotation of the chloroplast genome in *C. falcatum* was conducted using a Dual Organeller GenoMe Annotator (DOGMA) [22]. From this initial annotation, putative start and stop codons and intron positions were identified based on comparisons to homologous genes among genera *Cyrtomium*, *Woodwardia* [10] and *Pteridium* [23]. Identified transfer RNA (tRNA) genes were further confirmed using tRNAscan-SE 1.21 web server protocols [24]. A circle cp genome map was then drawn using the OrganellarGenomeDraw (OGDRAW) program (Max Planck Institute of Molecular Plant Physiology, Am Mühlenberg, Potsdam, Germany) [25].

### 2.3. Comparative Chloroplast Genomic Analysis

The complete cp genome of *C. falcatum* was compared with that of three other species, *C*. *devexiscapulae*, *Woodwardia unigemmata* and *Pteridium aquilinum*, using the mVISTA software program in Shuffle-LAGAN mode (Joint Genome Institute, United States Department of Energy, Walnut Creek, CA, USA) [26]. *C. falcatum* was set as the reference for these comparisons.

### 2.4. Analysis of Single Sequence Repeats 

PHOBOS v3.3.12 tandem repeat search software was used to identify single sequence repeats (SSRs). Analysis parameters of alignment scores for match, mismatch, gap, and N positions were set as 1, −5, −5, and 0, respectively (Animal Ecology, Evolution and Biodiversity, Ruhr-Universität Bochum, Bochum, Germany) [27].

### 2.5. Estimation of Substitution Rates

The *C. falcatum* cp genome sequence was compared with those of *C. devexiscapulae* [10], *W. unigemmata* [10] and *P. aquilinum* [23]. To analyze synonymous (K_S_) and non-synonymous (K_A_) substitution rates, the same individual functional protein-coding exons were extracted and aligned separately using the Geneious v7.1.9 bioinformatics software platform (Biomatters, Aukland, New Zealand). These aligned sequences were then translated into protein sequences and analyzed. Synonymous (K_S_) and non-synonymous (K_A_) substitution rates for each protein-coding exon were estimated using DNA Sequence Polymorphism (DnaSP) software (Evolutionary Genomics and Bioinformatics, Universitat de Barcelona, Gran Via de les Corts Catalanes, Barcelona) [28].

### 2.6. Analysis of RNA Editing

The online program, Plant RNA Editing—Prediction and Analysis Computer Tool (PREPACT) 2.0 (Institut für Zelluläre & Molekulare Botanik IZMB, Universität Bonn, Regina-Pacis-Weg, Bonn, Germany) for Plants [29], was used to identify possible RNA editing sites within the cp genome sequence of *C. falcatum*. For this analysis, the cut-off Expect (E) value was set as 0.001, minimal was set as 8, and filter threshold value was set as 70%.

### 2.7. Phylogenetic Analysis

In this study, the genome model was selected based on the close relationships between *C. falcatum* and other fern families. A molecular phylogenetic tree was constructed using 76 protein-coding genes from 23 different fern taxa. Among these, the genus *Ginkgo* was set as the outgroup. The 23 completed cp genome sequences representing various fern lineages were then downloaded from the National Center for Biotechnology Information (NCBI) Organelle Genome Resource database (Supplementary Table S1). The 76 protein-coding gene sequences were aligned using the MAFFT v7.017 (Computational Biology Research Center, The National Institute of Advanced Industrial Science and Technology, Tokyo, Japan) multiple sequence alignment program [30] via the Geneious v7.1.9 software platform. The aligned protein-coding gene sequences were saved using the Phylogeny Inference Package (PHYLIP) format in Clustal X v2.1 (The Conway Institute, University College Dublin, Dublin, Ireland) [31], and then used to generate a phylogenetic tree. Phylogenetic analysis was conducted based on maximum likelihood (ML) analysis using the general time-reversible invariant-sites nucleotide substitution model with default parameters in the Randomized Axelerated Maximum Likelihood (RAxML) v. 7.2.6 program (The Exelixis Lab, Scientific Computing Group, Heidelberg Institute for Theoretical Studies, Schloss-Wolfsbrunnenweg, Heidelberg, Germany) [32]. Bootstrap (BS) probability of each branch was calculated based on 1000 replications.

### 2.8. Molecular Clock Analysis

For divergence dating analyses, cp genomes were selected based on close relationships between *C. falcatum* and other fern families. A molecular clock tree was constructed using 76 protein-coding genes from 23 different fern taxa, and divergence dates were estimated using the Bayesian Evolutionary Analysis Sampling Trees (BEAST) v.2.1 software program (Centre for Computational Evolution, The University of Auckland, New Zealand) [33]. A strict clock model was implemented using Markov Chain Monte Carlo (MCMC) chains run for 20 million generations with 10% burn-in and sampled every 1000 generations. A GTR nucleotide substitution model was used with a gamma distribution with four rate categories. A Yule Process tree prior was used to estimate divergence times and creditability intervals. Sample size was evaluated using Tracer v.1.6 analysis software (Institute of Evolutionary Biology, University of Edinburgh, Edinburgh, Scotland) [34]. Tree data were summarized using TreeAnnotator v.2.1.2 (Centre for Computational Evolution, The University of Auckland, New Zealand) [33]. The calibration point was set for the divergence of the genus *Ginkgo* as 292.44–284.95 mya [35] and implemented a log normal distribution with a mean of 290.29 ± 28.34 mya.

## 3. Results

### 3.1. General Characteristics of the *Cyrtomium falcatum* cp Genome

**Figure 1:**
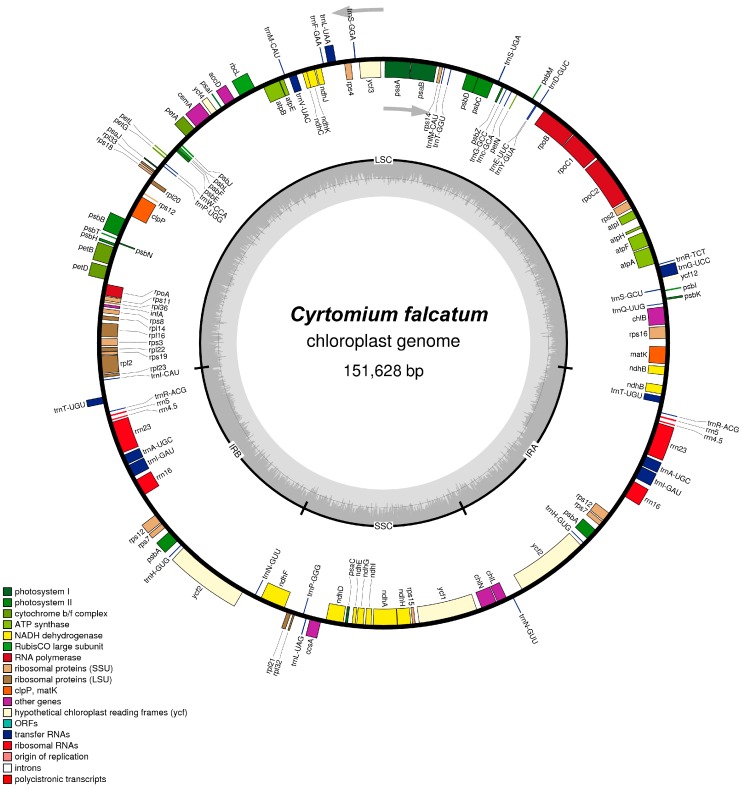
Gene map of *Cyrtomium falcatum*. Genes located on the outside of the outermost layer of the circle are transcribed in a counterclockwise direction, whereas genes located on the inside of the circle are transcribed in a clockwise direction. The colored bars indicate known protein-coding genes, transfer RNA (tRNA) genes and ribosomal RNA (rRNA) genes. The dashed, darker gray area in the inner circle denotes the guanine-cytosine (GC) content of the genome, whereas the lighter gray area indicates the adenosine-thymine (AT) content of the genome. Abbreviations annotated along the perimeter of the inner circle are translated as follows: LSC, large single copy; SSC, small single copy; IR, inverted repeat.

The total size of the complete *C. falcatum* genome was found to be 151,628 bp (KP189363). The genome demonstrated a characteristic circular structure with a pair of IRs of 23,852 bp separated by a large single copy (LSC) region of 82,308 bp and an SSC region of 21,616 bp (Figure 1). The guanine-cytosine (GC) content for the entire cp genome of *C. falcatum* was 42.3%. There were 131 individual genes identified, of which 116 genes were single copy, and 15 that were duplicated and occurred as inverted repeat sequences (Table 1). In terms of overall composition, there were four ribosomal RNA (rRNA) genes (6.1%), 34 individual tRNA genes (26.0%), 54 genes encoding photosynthesis-related proteins (41.2%), 24 genes encoding both large and small ribosomal subunit proteins (18.3%), four genes encoding DNA-dependent RNA polymerase subunits (3.1%), six genes encoding translation proteins (4.6%), and one gene encoding an unknown function (0.7%). Among the 116 single copy genes, 15 contained one intron and three genes encoded two introns (Table 2). Additionally, 22 functional genes possessed internal stop codons that indicated the presence of RNA editing sites. These internal stop codons were observed in all ferns.

### 3.2. Comparative Analysis of Genome Structure

**Figure 2:**
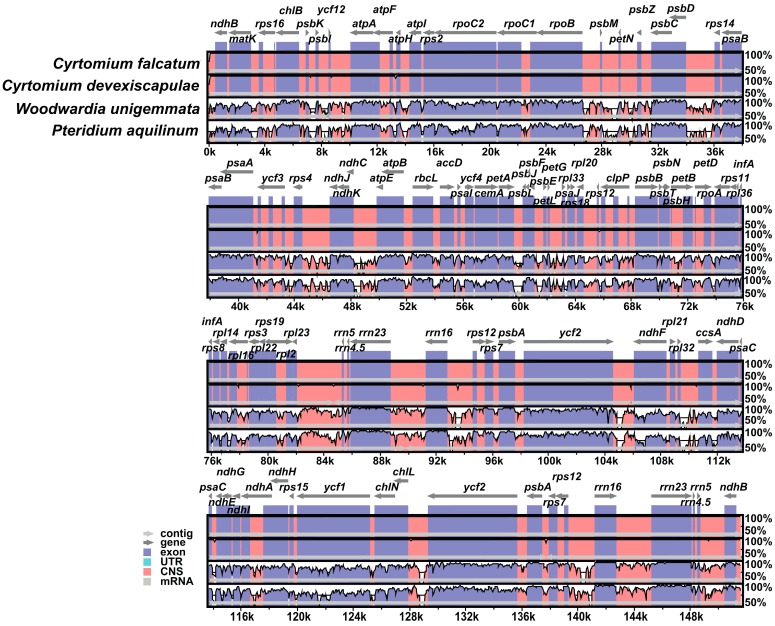
Comparison of the cp genome sequences of *C. falcatum*, *Cyrtomium devexiscapulae*, *Woodwardia unigemmata* and *Pteridium aquilinum* generated using mVISTA software. The gray arrows indicate the position and direction of each gene. The red and blue areas indicate intergenic and genic regions, respectively. Black lines define regions of sequence identity with *C. falcatum*, using a 50% identity cutoff. UTR: untranslated region; CNS: non-coding sequences.

The mVISTA software program set was used to study cp genome sequence variations in the order Polypodiales (Figure 2). The coding and non-coding regions of the genome were found to be more highly conserved in the Dryopteridaceae family. However, some dissimilarity was observed among the tRNA genes, e.g., the *trnR*-*UCG*, *trnR*-*CCG*, *trnSeC*, *trnV*-*GAC* and *trnV*-*GAU* genes were either deleted or were not present in the cp genome of *C. falcatum* (Figure 2).

Additionally, the start codons of 84 protein-coding genes were inferred using comparisons with previously annotated land plant cp genomes. Of the 84 protein genes present, 62 start with AUG, 20 start with ACG and two start with GUG (*ndhF* and *rps7*). Among these, four genes encoding the ACG start codon and two encoding the GUG start codon are located within the SSC region.

**Figure 3:**
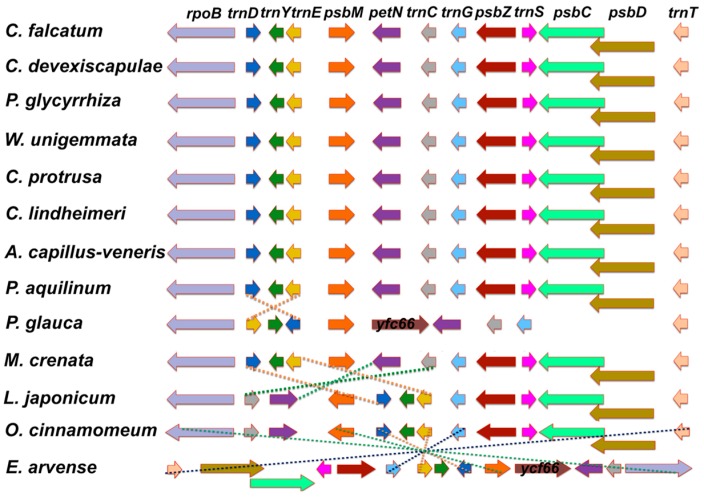
Gene organization from *rpoB* to *trnT* among analyzed ferns. The arrows correlate with the location, size and transcription direction of the corresponding genes. Dashed lines indicate putative local inversions within the corresponding cp genomes. Fern species are derived as follows: *C*. *falcatum: Cyrtomium falcatum*; *C*. *devexiscapulae: Cyrtomium devexiscapulae*; *P*. *glycyrrhiza: Polypodium glycyrrhiza*; *W*. *unigemmata: Woodwardia unigemmata*; *C*. *protrusa: Cystopteris protrusa*; *C*. *lindheimeri: Cheilanthes lindheimeri*; *A*. *capillus-veneris: Adiantum capillus-veneris*; *P*. *aquilinum: Pteridium aquilinum*; *P*. *glauca: Plagiogyria glauca*; *M*. *crenata: Marsilea crenata*; *L*. *japanicum: Lygodium japanicum*; *O*. *cinnamomeum: Osmundastrum cinnamomeum*; *E*. *arvense: Equisetum arvense*.

The significant inversions of the *C. falcatum* cp genome were compared with those of other ferns (Figure 3). Results indicated that the *C. falcatum* cp genome shared two significant inversions with other ferns, notably a minor inversion containing a single gene (*trnD*-*GUC*) and an approximately 3 kb inversion involving genes *trnG*, *psbZ*, *trnS*, *psbC*, *psbD,* and *trnT*.

### 3.3. Repeat Sequence Analysis

Distribution, type and presence of SSRs or microsatellites were analyzed in both the genic and intergenic regions of the *C. falcatum* cp genome. A total of 386 SSRs were identified (Table S2). Of these, 196 were distributed in the LSC regions, whereas 137 and 53 were located in the IR and SSC regions, respectively. Moreover, 163 SSRs were found in the protein-coding regions, 174 were located within intergenic spacers and 49 were found in the introns of the *C. falcatum* cp genome. Among these SSRs, mononucleotide repeats were found to be the most common, accounting for 48.96% of the total, whereas dinucleotide repeats accounted for 7%, trinucleotide repeats accounted for 10.36%, and tetra-, penta-, hexa-, 7-, 8-, 9-, 10-, 12-, 14-, 17-, and 24-nucleotide repeats occurred with less frequency. Additionally, 33 hexa-, eight 7-nucleotide, four 8-nucleotide, three 9-nucleotide, and one each 10-, 12-, 13-, 14-, 17-, and 24-nucleotide repeats were detected in the cp genome. The size and location of hexa-, 7-, 8-, 9-, 10-, 12-, 13-, 14-, 17-, and 24-nucleotide repeats are shown in Table S3. A total of 54 repeats (>12 bp) were identified within the genome, whereas 28 were localized within intergenic spacers, 19 were located in coding regions and seven were found in introns.

### 3.4. Synonymous and Non-Synonymous Substitution Rate Analysis

**Figure 4:**
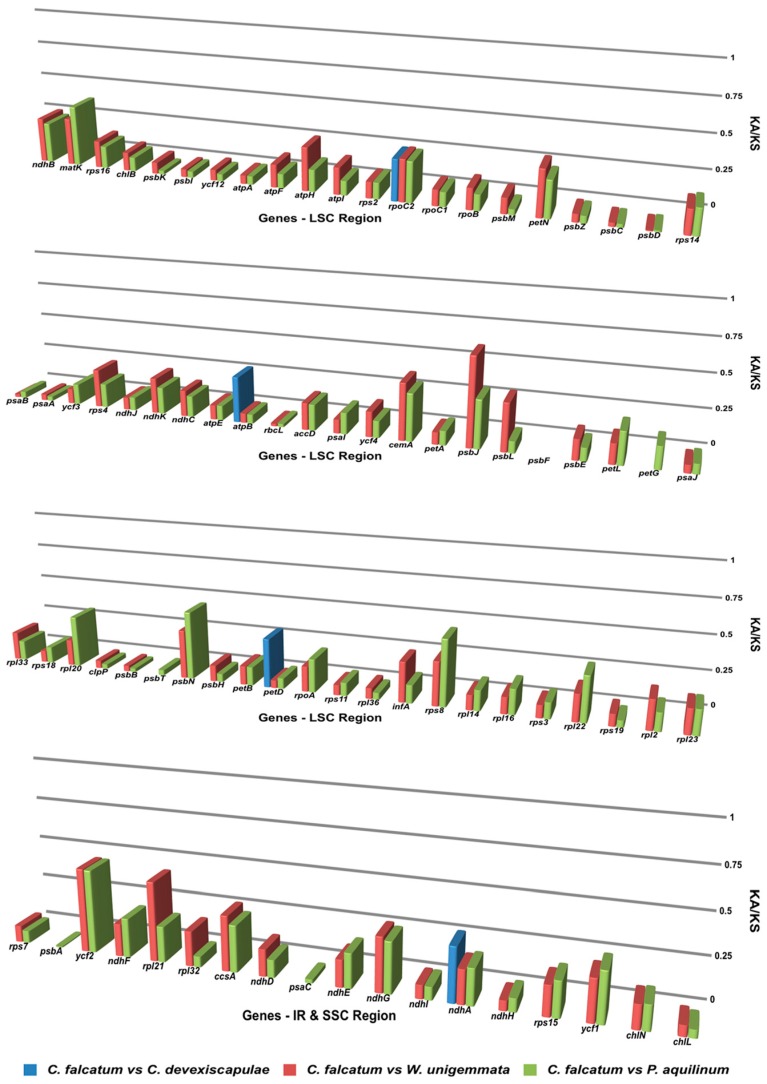
Synonymous/non-synonymous (K_A_/K_S_) substitution rate values for the 76 protein-coding genes of *C. falcatum*, *C. devexiscapulae*, *W. unigemmata* and *P. aquilinum*. The blue boxes indicate the K_A_/K_S_ ratio of *C. falcatum* compared to *C. devexiscapulae*, the red boxes indicate the K_A_/K_S_ ratio of *C. falcatum* compared to *W. unigemmata* and the green boxes indicate the K_A_/K_S_ ratio of *C. falcatum* compared to *P. aquilinum*.

The synonymous and non-synonymous substitution rates for *C. falcatum* were compared with those of closely related species *C. devexiscapulae* [10], *W. unigemmata* [10] and *P. aquilinum* [22]; results indicated that these rates were less than one for all genes (Figure 4). Among these findings, the gene *petD* of *C. falcatum*, compared to that of *C. devexiscapulae*, demonstrated the highest K_A_/K_S_ ratio at 0.354, whereas the gene *ycf2* of *C. falcatum,* when compared with both *W. unigemmata* and *P. aquilinum*, indicated the highest K_A_/K_S_ ratios of 0.502 and 0.498, respectively.

### 3.5. RNA Editing of *Cyrtomium* cp Genomes

**Figure 5:**
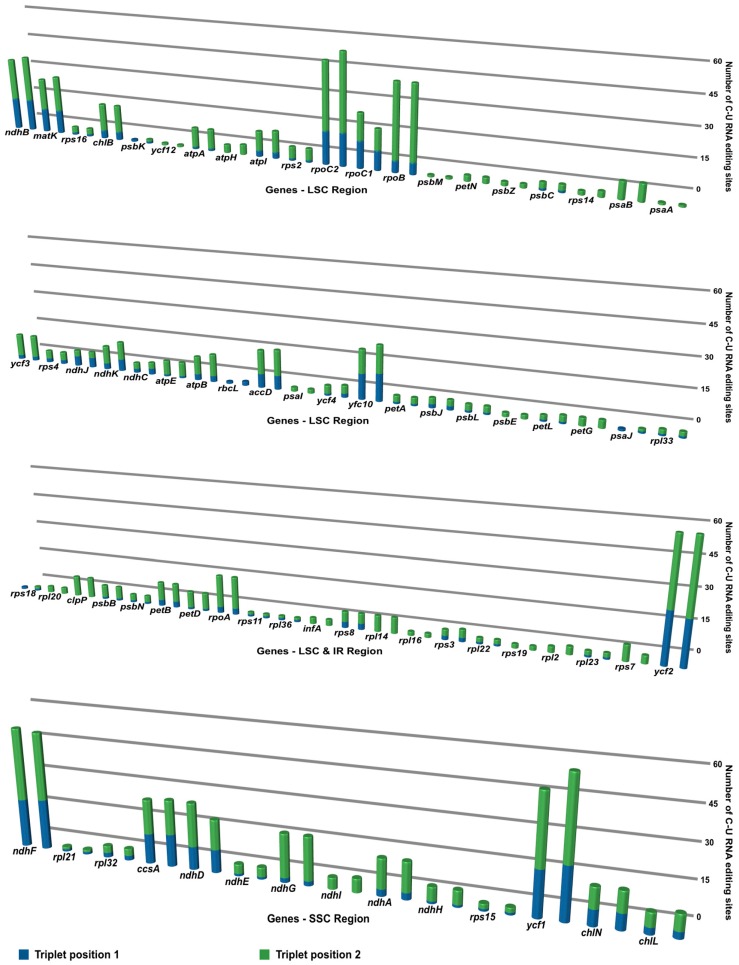
Triplet position and number of C-to-U RNA editing sites in plastid genes of *C. falcatum* and *C. devexiscapulae* were compared. Columns represent the protein-coding genes of *C. falcatum* and *C. devexiscapulae*, respectively. The blue cylindrical column indicates C-to-U RNA editing events that occurred in triplet position 1, whereas the green cylindrical column indicates C-to-U RNA editing events that occurred in triplet position 2.

**Figure 6:**
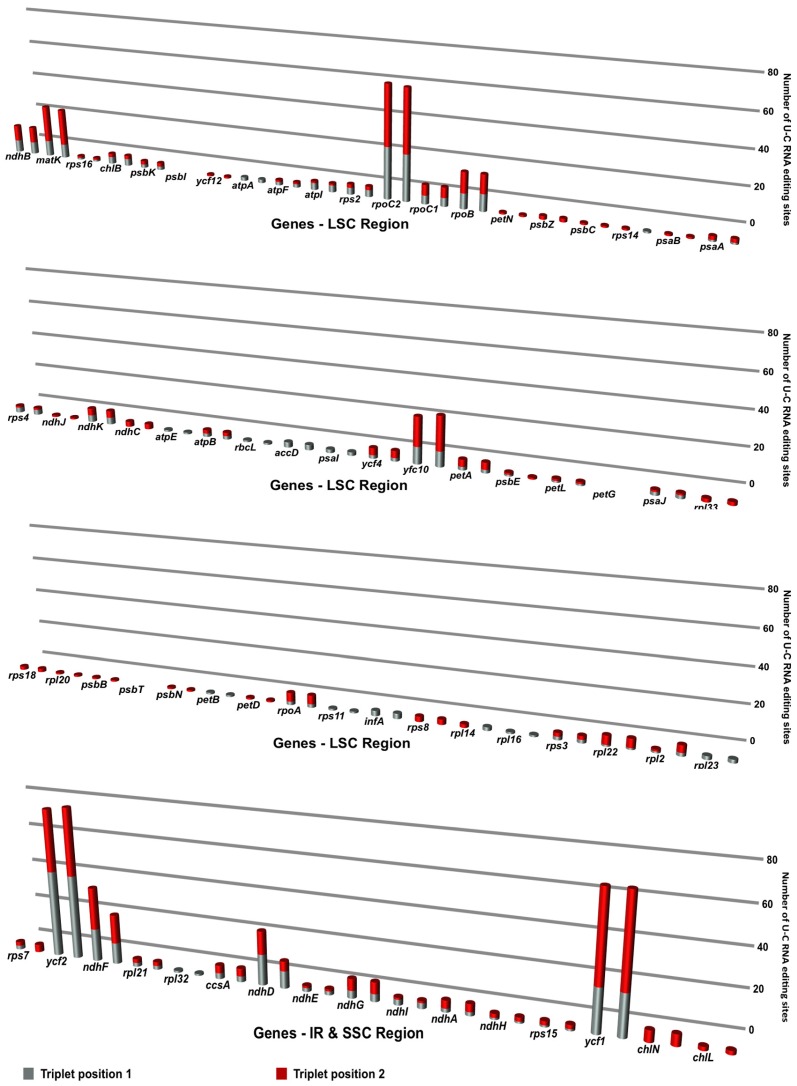
Triplet position and number of U-to-C RNA editing sites in plastid genes of *C. falcatum* and *C. devexiscapulae* were compared. Columns represent the protein-coding genes of *C. falcatum* and *C. devexiscapulae*, respectively. The gray cylindrical column indicates U-to-C RNA editing events that occurred in triplet position 1, whereas the red cylindrical column indicates U-to-C RNA editing events that occurred in triplet position 2.

The RNA editing events were analyzed in all plastid protein-coding genes in *C. falcatum* and *C. devexiscapulae*. The C-to-U (Figure 5) and U-to-C (Figure 6) RNA editing events were calculated separately at triplet positions 1 and 2, and then compared to both cp genomes of *Cyrtomium*. Results indicated that the number of these two RNA editing events varied between the two cp genomes. The protein-coding genes in the *C. falcatum* cp genome revealed RNA editing at 841 sites in the form of C-to-U conversions (Figure 5) and at 562 sites in the form of U-to-C conversions (Figure 6), whereas 867 C-to-U and 537 U-to-C RNA editing sites were observed in *C. devexiscapulae*.

**Figure 7:**
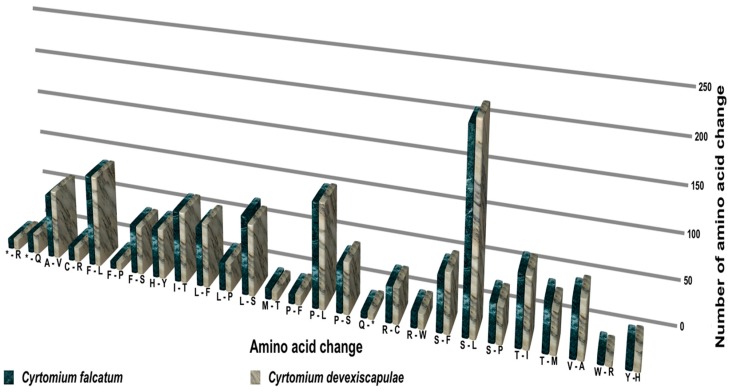
The number of amino acid changes in the cp genomes of *C. falcatum* and *C. devexiscapulae*. The green column indicates the amino acid changes that occurred in the protein-coding genes of *C. falcatum*, whereas the gray column indicates the amino acid changes that occurred in the protein-coding genes of *C. devexiscapulae*.

Closely related genomes typically contain regions encoding closely related proteins. Alignments in sequences of homologous genes reveal some differences, mostly in the form of single-site mutations or indels. There is often a reasonable correlation between overall species divergence and the divergence of sequences of individual genes and their corresponding proteins. In this study, we compared amino acid changes in the protein-coding genes of *C. falcatum* with closely related *C. devexiscapulae*. A total of 1478 and 1494 amino acids within the protein-coding regions of *C. falcatum* and *C. devexiscapulae*, respectively, were changed into other amino acids by RNA editing (Figure 7). Among these, 222 amino acids (15.02%) changed from serine to leucine, 117 (7.92%) changed from proline to leucine and 103 (6.97%) changed from phenylalanine to leucine.

### 3.6. Phylogenetic Analysis

**Figure 8:**
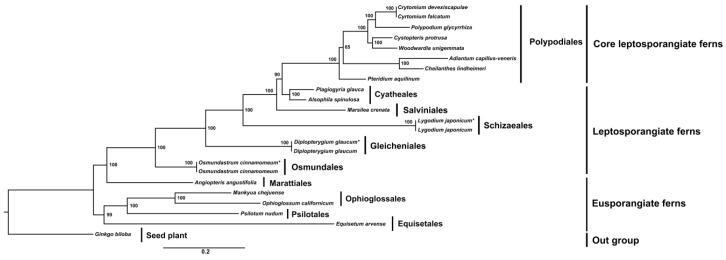
Molecular phylogenetic tree of 23 distinct fern taxa based on 76 protein-coding genes in the cp genome. The tree was constructed by maximum likelihood (ML) analysis of conserved regions using the Randomized Axelerated Maximum Likelihood (RAxML) program and the general time-reversible invariant-sites nucleotide substitution model. The stability of each tree node was tested by bootstrap analysis using 1000 replicates. Bootstrap values are indicated on each of the branches, whereas the branch length reflects the estimated number of substitutions per 1000 sites. The genus *Ginkgo* was set as the outgroup. * Fossil data cp genomes were used to identify phylogenetic relationships with corresponding taxa.

In this study, we investigated the relationship between eupolypod, leptosporangiate and eusporangiate ferns. We have compared the chloroplast genes of Dokdo populations with other populations from Japan (*rbcL*, AB575102), Taiwan (*matK*, JF303945) and USA (*atpA*, EF463671). However, all sequences showed 100% homology with Dokdo populations and no dissimilarity has been observed. Eusporangiate ferns are grouped in four clades: Psilotales, Ophioglossales, Equisetales and Marattiales. Phylogenetic analysis of fern cp genomes strongly supported monophyly with a 100% BS value (Figure 8). *Equisetum* was identified as a sister genus to *Psilotum*, *Ophioglossum* and *Mankyua* with strong BS support of 100%. The clade of marattioid ferns formed a sister clade to the leptosporangiate ferns with 100% BS support. Among the core leptosporangiate ferns, *Marsilea* formed a sister relationship between tree ferns and polypods that was strongly supported by 76 protein-coding genes with a 100% BS value. Dennstaedtiaceae was also suggested to be a sister to the Pteridaceae clade and the eupolypods according to a moderate BS value of 65%. However, the sister relationship between Pteridaceae and eupolypods was strongly supported with a 100% BS value.

### 3.7. Divergence Dating Analysis

**Figure 9:**
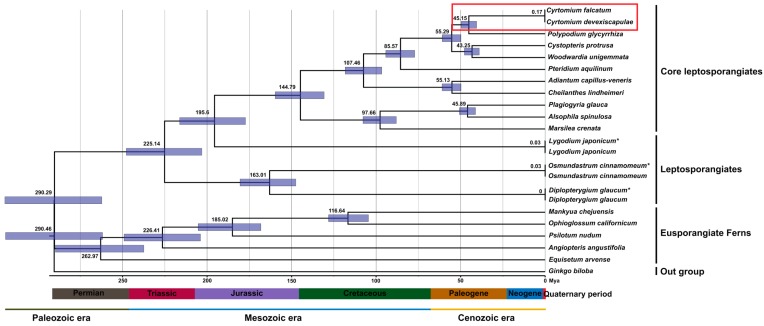
The molecular clock was constructed based on 76 protein-coding genes of 23 different fern taxa cp genomes using the Bayesian Evolutionary Analysis Sampling Trees (BEAST) software program. Mean age estimates (in millions of years) are shown along the branches. The gray bars represent the 95% posterior density credibility interval for node ages. * Fossil data cp genomes were used to identify the molecular age of the corresponding taxa.

In this study, we analyzed the divergence time of *C. falcatum* to determine its origin. Historically, the genus *Ginkgo* diversified in the late Permian period (290.29 ± 28.32 mya) (Figure 9), whereas among fern groups, eusporangiate ferns diversified in the mid-Permian (262.97 ± 28.18 mya) to late Cretaceous (116.64 ± 12.08 mya). Leptosporangiates diversified in the middle Triassic (225.14 ± 21.69 mya) to early Jurassic (163.01 ± 18.88 mya) periods, whereas the leptosporangiate core diversified between the early Triassic (144.79 ± 15.46 mya) and mid-Paleogene (43.25 ± 4.57 mya) periods. In contrast, eupolypods diversified in the late Paleogene (55.29 ± 5.36 mya) period. Within this group, the genera *Cyrtomium* and *Polypodium* diversified first (45.15 ± 4.93 mya), whereas *Cystopteris* and *Woodwardia* diversified later (43.25 ± 4.57 mya). Nevertheless, Bayesian molecular clock analyses suggest that the fern plant *C. falcatum* diversified in the middle of the Paleogene period, approximately 45.15 ± 4.93 mya.

## 4. Discussion

### 4.1. General Characteristics of the *Cyrtomium falcatum* cp Genome

Islands are ideal model systems for evolutionary studies. Dokdo Island is located between the Korean Peninsula and Japan, and is home to 49 different plant species [36], including only a single fern plant, *C*. *falcatum*. To date, no evolutionary studies of fern plants have been conducted on Dokdo Island using chloroplast (cp) genomes. Therefore, we sought to understand the molecular phylogenetic and evolutionary relationships of this fern plant from Dokdo Island. In this study, the total size of the *C. falcatum* genome was found to be 151,628 bp (KP189363), which is approximately the same size as that of *C. devexiscapulae* (151,684 bp) [10]. The cp genome of *C. falcatum* is smaller than those of *P. aquilinum* (152,362 bp) [23], *W. unigemmata* (153,717 bp) [10] and *C. lindheimeri* (155,770 bp) [37], but larger than that of *A. capillus*-*veneris* (150,568 bp) [12]. Such variation in cp genome size results from the expansion and contraction of the border areas between IR regions and single copy regions. The *C. falcatum* cp genome was also found to have an AT content of 57.7%, which is identical to that of *C. devexiscapulae* (57.7%), higher than those of *W. unigemmata* (56.8%) and *C. lindheimeri* (57.3%), but smaller than those of *A. capillus*-*veneris* (58%) and *P. aquilinum* (58.5%). This variation in AT content might be due to the presence of a non-coding region in the core leptosporangiate cp genome. In general, however, the gene content and gene order in the *C. falcatum* cp genome is virtually identical to that of *C. devexiscapulae*.

### 4.2. Comparative Analysis of Genome Structure

mVISTA software was used to study cp genome sequence variations among members of the order Polypodiales (Figure 2). Coding and non-coding regions were highly conserved in the Dryopteridaceae family, whereas the greatest dissimilarity was observed in the cp genomes of *W. unigemmata* and *P. aquilinum* when compared with cp genomes of ferns belonging to other families. Interestingly, the tRNA genes *trnR*-*UCG*, *trnR*-*CCG* and *trnSeC* were not present in the cp genome of *C. falcatum* or other eupolypod ferns. However, the *trnR*-*UCG* gene was present in the *Alsophila* cp genome [38], the *trnR*-*CCG* gene was found in non-flowering land plants, including *Angiopteris* and *Psilotum* [6], and the *trnSeC* gene was present in the *Adiantum* cp genome [12]. These results suggest that these genes are restricted only to tree ferns, non-core leptosporangiates and basal ferns. Sugiura and Sugita [39] also reported that the *trnR*-*CCG* gene is not essential for plastid function, even though it is highly conserved in the chloroplasts of non-flowering plants.

Interestingly, the start codon GUG of genes *ndhF* and *rps7* is only found in polypod ferns. Other groups of ferns, such as tree ferns, heterosporous ferns and leptosporangiate ferns, encoded the AUG initiation codon for these two genes. In contrast, Gao et al. [7] reported that the *psbC* and *rps12* genes encoded the GUG initiation codon in the cp genome of the tree fern *Alsophila*.

The *C. falcatum* cp genome shared two significant inversions with other ferns (Figure 3), a minor inversion near *trnD*-*GUC* and an approximately 3 kb inversion. These two inversions are restricted to ferns; however, cp genomes of other tree ferns such as *Alsophila* share another 30 kb inversion at the beginning of the LSC region that is also observed in all vascular plants except for lycophytes [7]. Additionally, three conserved and consecutive tRNA genes, *trnD*-*GUC*, *trnY*-*GUA* and *trnE*-*UUC*, have been identified in all cp genomes of land plants. These three genes have the same transcription directions found in all land plants except ferns. In ferns, the *trnD* gene is inverted relative to genes *trnY* and *trnE*. This may be due to a single minor inversion in the *trnD* gene. The 3 kb inversion appears to be identical to that of other ferns such as *C. devexiscapulae*, *W. unigemmata*, *C. lindheimeri* and *A. capillus*-*veneris*, whereas *Angiopteris* and *Psilotum* follow the *Osmunda* gene order.

### 4.3. Repeat Sequence Analysis

Chloroplast genomes of land plants have highly conserved structures and content organization. Similar regions with high sequence polymorphisms are commonly observed in closely related species [40]. A total of 386 SSRs were identified in the genic and intergenic regions of the *C. falcatum* cp genome. Mono, di-, tetra-, penta-, 7-, 8-, 9-, 10-, 12-, 14-, 17-, and 24-nucleotide repeats were observed in the intergenic regions. Comparatively, the genic regions had more tri- and hexa-nucleotide SSRs; the implications of this could be extremely important for genomic stability and gene functions within the cp genome. However, the presence of several SSR sites in the cp genome indicates that these sites could be assessed for intraspecific levels of polymorphism, leading to highly sensitive phylogeographic and population structure studies for this species.

### 4.4. Synonymous and Non-Synonymous Substitution Rate Analysis

Nucleotide substitution rates are very important indicators in gene evolution studies [41,42,43]. Makalowski and Boguski [44] stated that the ratio of K_A_/K_S_ was less than one in most protein-coding genes. However, non-synonymous substitution rates occurred less frequently than their synonymous counterparts. The K_A_/K_S_ rates of the *C. falcatum* cp genome were compared with those of closely related species. The results suggested that the rates of synonymous and non-synonymous substitutions did not vary among genes of closely related species. Therefore, all of these genomes contain similar sets of genes, and their cp genomes evolved in similar ways.

### 4.5. RNA Editing of Cyrtomium cp Genomes

RNA editing modifies genetic information at the transcript level, and primarily occurs in the mitochondrial and cp genomes of higher plants [45]. During these events, substitutions or indel mutations may occur, possibly leading to alternations in the transcription process. The majority of C-to-U and U-to-C RNA editing sites occur in protein-coding genes *ndhF*, *rpoB*, *rpoC2*, *ycf1*, and *ycf2*, with over 40 RNA editing events modifying start or stop codons. Previous studies indicated that most seed plant plastomes have about 30–40 RNA editing sites [46]. Wolf et al. [36] reported that RNA editing was highest in hornworts (approximately 972 sites) and intermediate in the fern *Adiantum* (approximately 344 sites). Most RNA editing involved C-to-U conversions rather than U-to-C by base modification [47]. RNA editing generally occurs in the mitochondrial genome, but this phenomenon is significantly less frequent in chloroplasts [48]. Nevertheless, the total number of C-to-U RNA editing sites in the first and second positions is 255 and 586 in the cp genome of *C. falcatum*, and 259 and 608 in that of *C. devexiscapulae*, respectively. Conversely, the total number of U-to-C RNA editing sites in triplet positions 1 and 2 is 250 and 312 in *C. falcatum*, and 222 and 315 in *C. devexiscapulae*, respectively. These results revealed that the RNA editing rate was very high at the second triplet position compared to the first position, and that it differed from that of *Adiantum*. However, the results of this study suggest that levels of RNA editing may vary greatly within each of these large clades of plants. When compared with other amino acids, the conversion rate of amino acids to leucine is very high. Previous studies also reported that RNA editing of C/U at the second codon position has occurred mainly in plant organelles to increase the frequency of the highly hydrophobic amino acid leucine [49]. Mungpakdee et al. [50] suggested that nine types of RNA editing have occurred in *Symbiodinium* to increase molecular hydropathy. Thus, similar environments within organelles may force RNA editing to produce similar outcomes.

### 4.6. Phylogenetic Analysis

The phylogenetic relationships among Monilophytes, including four orders of eusporangiate ferns (Psilotales, Ophioglossales, Equisetales and Marattiales), as well as a clade of leptosporangiate ferns orders, were unclear, although recent studies [6,11,15,35,51,52,53] showed that they constitute a paraphyletic assemblage. However, phylogenetic analyses based on chloroplast genomes confirmed both the paraphyly of eusporangiate ferns and the sister relationship between Marattiales and leptosporangiate ferns [6,10,53,54]. In a recent study, Lu et al. [10] revealed that marattioid ferns were sisters to leptosporangiate ferns. In our study, *Equisetum* formed a sister clade to Psilotum, Ophioglossum and Mankyua with strong BS support (100%). However, in a previous study, Zhong et al. [53] reported that placement of *Equisetum* in various analyses did not have strong statistical support, and when the cp genome of *Tmesipteris bernh*. (another genus of Psilotaceae) was added to these phylogenetic analyses, *Equisetum* was found to be a sister to Psilotaceae with strong BS support. In our study, we did not use any other genera of Psilotaceae; nevertheless, we found that Equisetales was a sister to Psilotales-Ophioglossales with strong support. Moreover, the results of our study strongly support the relationship of an Equisetales sister with the Psilotales-Ophioglossales clade as in Lu et al. [10]. The clade of marattioid ferns formed a sister clade to the leptosporangiate ferns with a 100% BS value, whereas in the core leptosporangiate ferns, *Marsilea* formed a sister relationship between tree ferns and polypods that was strongly supported by 76 protein-coding genes with a 100% BS value. Dennstaedtiaceae was also a sister to the Pteridaceae clade and eupolypods with a moderate BS value of 65%. However, the sister relationship between Pteridaceae and eupolypods was strongly supported by a 100% BS value. In contrast, Lu et al. [10] reported that Pteridaceae was a sister to the Dennstaedtiaceae clade and eupolypods with high support (100% BS), and a sister relationship between Dennstaedtiaceae and eupolypods was strongly supported (98% BS). In their study, they used two different sets of cp genes consisting of 48 and 64 genes, respectively. In our study, we used 76 protein-coding genes from 23 taxa for this analysis. Thus, variation in BS values may be due to the number of genes used in this study. Lu et al. [10] suggested that a 48-gene dataset showed higher support values than a 64-gene dataset. Therefore, to confirm the position of Pteridaceae, it will be necessary to expand taxon sampling as the next step in future phylogenomic analysis of polypods.

### 4.7. Divergence Dating Analysis

The major lineages of the eusporangiate ferns, leptosporangiate ferns, polypod ferns and tree ferns originated in the mid-Permian, mid-Triassic, and middle and late Cretaceous periods, respectively. All extant members of *Equisetum* diversified only within the last 50 million years, whereas Equisetales was estimated to have a Paleozoic origin [55]. Our study strongly supports the hypothesis that *E. arvense* diversified during the Paleozoic era (262.97 ± 28.18 mya), and *Pteridium aquilinum* diversified during the early Cretaceous period (85.57 ± 8.25 mya). The earliest divergences of eupolypods within each of these lineages occurred 55.29 ± 5.36 mya. The order Polypodiales diversified 45.15 ± 4.93 mya, whereas Dryopteridales and Blechnales diversified in the middle of the Paleogene period (43.25 ± 4.57 mya). Celaya and McCabe [56] reported that Japan separated from the Eurasian continent approximately 15 mya, the East Sea formed in the early Miocene epoch (15 to 12 mya), but Dokdo Island formed later, in the mid-Pleistocene epoch (4.6 to 2.5 mya) [19]. *C. falcatum* is found on the Korean Peninsula, and Japan and its islands, but is the only fern plant present on Dokdo Island. In this study, we analyzed the divergence time of *C. falcatum* to determine its period of origin. Bayesian molecular clock analyses suggest that this fern plant diversified in the mid-Paleogene period (45.15 ± 4.93 mya). Moreover, the *Cyrtomium* plant lineage diverged very recently, compared to other eupolypods (55.29 ± 5.36 mya). Based on this divergent time analysis, we estimate that *C. falcatum* may have moved from Eurasia to Dokdo Island, and Japan and its islands, prior to island separation from the mainland.

## 5. Conclusions

In summary, we present the complete cp genome sequence of *Cyrtomium falcatum*, a holly fern, and provide a comprehensive comparative analysis of the cp genomes of eupolypod ferns. The cp genomes of the two characterized *Cyrtomium* species are very similar in terms of both gene content and gene order; therefore, they provided little information regarding the complexity of the fern cp genome. However, some dissimilarities were observed in the fern cp genomes, including the encoding of GUG as a start codon in genes *ndhF* and *rps7*, which is only found in polypod ferns. Similarly, genes *trnV*-*GAC* and *trnV*-*GAU* were deleted from the *C. falcatum* genome, whereas genes *trnP*-*GGG* and *rpl21* were identified in *C. falcatum*. These findings suggest that these genes are not conserved in all angiosperm cp genomes. Moreover, the tRNA genes, *trnR*-*UCG*, *trnR*-*CCG* and *trnSeC*, were not present in the cp genome of *C. falcatum* or other eupolypod ferns, indicating that these genes are probably restricted to tree ferns, non-core leptosporangiates and basal ferns. The *C. falcatum* cp genome also shares two significant inversions with other ferns, including a small inversion of the *trnD*-*GUC* region and an approximately 3 kb inversion of the *trnG-trnT* region. There were 76 protein-coding sequences from 23 fern species employed to construct phylogenetic trees, providing strong support for a monophyletic group in the fern clade. *Equisetum* was basal to eusporangiate ferns, with a strong BS value (100%), whereas Dennstaedtiaceae was basal to eupolypods with a moderate BS value (65%). Bayesian molecular clock analyses suggested that *C. falcatum* diversified in the mid-Paleogene period (45.15 ± 4.93 mya), and showed that this species may have moved from the Eurasian continent to Dokdo Island before the island separated from the mainland. Overall, the results of this study contribute to a better understanding of the genome structure of eupolypod ferns and their phylogenetic relationships with other fern taxa. However, confirmation of the position of the Pteridaceae is still needed; thus, expanded taxon sampling should be conducted as the next step in the phylogenomic analysis of polypods.

## Figures and Tables

**Table 1:** List of genes present in the chloroplast (cp) genome of *Cyrtomium falcatum.*

Category	Group of Genes	Name of Genes				
RNA genes	Ribosomal RNA genes	*rrn4.5* ^a^	*rrn5* ^a^	*rrn16* ^a^	*rrn23* ^a^	
	Transfer RNA genes	*trnA-UGC* ^a,b^	*trnC-GCA*	*trnD*-*GUC*	*trnE*-*UUC*	*trnF*-*GAA*
		*trnfM*-*CAU*	*trnG-GCC*	*trnG*-*UCC* ^b^	*trnH*-*GUG* ^a^	*trnI*-*CAU*
		*trnI*-*GAU* ^a,b^	*trnL*-*UAA* ^b^	*trnL*-*UAG*	*trnM*-*CAU*	*trnN*-*GUU* ^a^
		*trnP*-*GGG*	*trnP-UGG*	*trnQ*-*UUG*	*trnR*-*ACG* ^a^	*trnR*-*UCU*
		*trnS*-*GCU*	*trnS-GGA*	*trnS*-*UGA*	*trnT*-*GGU*	*trnT*-*UGU* ^a,b^
		*trnV*-*UAC* ^b^	*trnW* *-CCA*	*trnY*-*GUA*		
Protein genes	Subunits of photosystem I	*psaA*	*psaB*	*psaC*	*psaI*	*psaJ*
		*ycf3* ^c^	*ycf4*			
	Subunits of photosystem II	*psbA* ^a^	*psbB*	*psbC*	*psbD*	*psbE*
		*psbF*	*psbH*	*psbI*	*psbJ*	*psbK*
		*psbL*	*psbM*	*psbN*	*psbT*	*psbZ*
		*ycf12*				
	Subunits of cytochrome	*petA*	*petB* ^b^	*petD* ^b^	*petG*	*petL*
		*petN*				
	Subunits of ATP synthase	*atpA*	*atpB*	*atpE*	*atpF* ^b^	*atpH*
		*atpI*				
	Large subunit of RuBisCO	*rbcL*				
	Subunits of NADH dehydrogenase	*ndhA* ^b^	*ndhB* ^a,b^	*ndhC*	*ndhD*	*ndhE*
		*ndhF*	*ndhG*	*ndhH*	*ndhI*	*ndhJ*
		*ndhK*				
	ATP-dependent protease subunit P	*clpP* ^c^				
	Chloroplast envelope membrane protein	*cemA*				
	Light-independent Pchlide oxidoreductase (DPOR)	*chlB*	*chlL*	*chlN*		
Ribosomal proteins	Small subunit of ribosome	*rps2*	*rps3*	*rps4*	*rps7* ^a^	*rps8*
		*rps11*	*rps12* ^a,c,d^	*rps14*	*rps15*	*rps16* ^b^
		*rps18*	*rps19*			
	Large subunit of ribosome	*rpl2*	*rpl14*	*rpl16* ^b^	*rpl20*	*rpl21*
		*rpl22*	*rpl23*	*rpl32*	*rpl33*	*rpl36*
Transcription	DNA-dependent RNA polymerase	*rpoA*	*rpoB*	*rpoC1* ^b^	*rpoC2*	
Translation	Translational initiation factor	*infA*				
Other proteins	Maturase	*matK*				
	Subunit of acetyl-CoA	*accD*				
	C-type cytochrome synthesis gene	*ccsA*				
	Component of TIC complex	*ycf1*				
	Hypothetical proteins	*ycf2* ^a^				

^a^ Two gene copies in inverted repeats (IRs); ^b^ Gene containing a single intron; ^c^ Gene containing two introns; ^d^ Gene divided into two independent transcription units.

**Table 2:** Location and length of intron-containing genes within the *C. falcatum* chloroplast genome.

Gene *	Location	Exon I	Intron I	Exon II	Intron II	Exon III
		Nucleotides in Base Pairs				
*atpF*	LSC	145	720	410		
*clpP*	LSC	240	571 7	292	723	71
*ndhA*	SSC	561	972	555		
*ndhB*	IR	780	874	498		
*petB*	LSC	6	848	642		
*petD*	LSC	8	643	472		
*rps12 ^#^*	LSC	114	--	232	577	26
*rpl2*	IR	397	725	437		
*rpl16*	LSC	9	1068	399		
*rpoC1*	LSC	433	696	1610		
*rps16*	LSC	9	795	405		
*trnA-UGC*	IR	36	798	37		
*trnG-UCC*	LSC	23	907	48		
*trnI-GAU*	IR	36	1016	36		
*trnL-UAA*	LSC	34	637	51		
*trnT-UGU*	IR	34	507	40		
*trnV-UAC*	LSC	40	618	34		
*ycf3*	LSC	125	625	229	725	162

* Identical, duplicate genes containing introns in the IR region are not included; **^#^** *rps12* is a trans-spliced gene with the 5′ end located in the large single copy (LSC) region; it is duplicated in the 3′ end in the IR regions.

## Supplementary Materials

The following are available online at www.mdpi.com/2073-4425/7/12/115/s1, Table S1: Accession numbers of the chloroplast genome sequences used in this study, Table S2: List of identified simple sequence repeats of *Cyrtomium falcatum* chloroplast genome, Table S3: Distribution of hexa-, 7-, 8-, 9-, 10-, 12-, 13-, 14-, 17-, and 24-nucleotide single sequence repeats (SSRs) in *Cyrtomium falcatum* chloroplast genome.
