# Supplementary material for: Phylogenetic Relationships of the Fern Cyrtomium falcatum (Dryopteridaceae) from Dokdo Island Based on Chloroplast Genome Sequencing
Source: Genes (Basel). 2016 Dec 2;7(12):115. doi: 10.3390/genes7120115 (PMC5192491; doi:10.3390/genes7120115)
Supplement: Supplementary file 1 [file genes-07-00115-s001.docx]

Supplementary Materials: Phylogenetic Relationships of the Fern *Cyrtomium falcatum* (Dryopteridaceae) from Dokdo Island, Sea of East Japan, Based on Chloroplast Genome Sequencing

Gurusamy Raman, Kyoung Su Choi and SeonJoo Park

**Table S1.** The list of accession numbers of the chloroplast genome sequences was used in this study.

| **Sl. No.** | **Taxon** | **Family** | **Order** | **GenBank** |
| --- | --- | --- | --- | --- |
| 1 | *Adiantum capillus-veneris* | Pteridaceae | Polypodiales | NC_004766 |
| 2 | *Alsophila spinulosa* | Cyatheaceae | Cyatheales | NC_012818 |
| 3 | *Angiopteris angustifolia* | Marattiaceae | Marattiales | NC_026300 |
| 4 | *Cheilanthes lindheimeri* | Pteridaceae | Polypodiales | NC_014592 |
| 5 | *Cyrotomium devexiscapulae* | Dryopteridaceae | Polypodiales | KT599100 |
| 6 | *Cyrtomium falcatum* | Dryopteridaceae | Polypodiales | KP189363 |
| 7 | *Cystopteris protrusa* | Cystopteridaceae | Polypodiales | KP136830 |
| 8 | *Diplopterygium glaucum* * | Gleicheniaceae | Gleicheniales | NC_024158 |
| 9 | *Diplopterygium glaucum* | Gleicheniaceae | Gleicheniales | KF225594 |
| 10 | *Equisetum arvense* | Equisetaceae | Equisetales | NC_014699 |
| 11 | *Ginkgo biloba* | Ginkgoaceae | Ginkgoales | AB684440 |
| 12 | *Lygodium Japanicum* * | Lygodiaceae | Schizaeales | NC_022136 |
| 13 | *Lygodium Japanicum* | Lygodiaceae | Schizaeales | KF225593 |
| 14 | *Mankyua chejuensis* | Ophioglossaceae | Ophioglossales | NC_017006 |
| 15 | *Marsilea crenata* | Marsileaceae | Salviniales | NC_022137 |
| 16 | *Ophioglossum californicum* | Ophioglossaceae | Ophioglossales | NC_020147 |
| 17 | *Osmundastrum cinnamomeum* * | Osmundaceae | Osmundales | NC_024157 |
| 18 | *Osmundastrum cinnamomeum* | Osmundaceae | Osmundales | KF225592 |
| 19 | *Plagiogyria glauca* | Plagiogyriaceae | Cyatheales | KP136831 |
| 20 | *Polypodium glycyrrhiza* | Polypodiaceae | Polypodiales | KP136832 |
| 21 | *Psilotum nudum* | Psilotaceae | Psilotales | NC_003386 |
| 22 | *Pteridium aquilinum* | Dennstaedtiaceae | Polypodiales | NC_014348 |
| 23 | *Woodwardia unigemmata* | Blechnaceae | Polypodiales | KT599101 |

* Fossil data were used.

**Table S2.** List of identified simple sequence repeats of *Cyrtomium falcatum* chloroplast genome.

| **SSR Sequence** | **Number of Repeats** | | | | | | | | | | | | | | | | |
| --- | --- | --- | --- | --- | --- | --- | --- | --- | --- | --- | --- | --- | --- | --- | --- | --- | --- |
|  | 2 | 3 | 4 | 5 | 6 | 7 | 8 | 9 | 10 | 11 | 12 | 13 | 14 | 15 | 16 | 17 | Total |
| A/T |  |  |  |  |  | 69 | 14 | 15 | 14 | 3 | 2 | 2 | 2 |  |  |  | 121 |
| G/C |  |  |  |  |  | 23 | 9 | 5 | 14 | 8 | 2 |  | 1 | 4 | 1 | 1 | 68 |
| AC/GT |  |  | 1 | 1 |  |  |  |  |  |  |  |  |  |  |  |  | 2 |
| AG/CT |  |  | 15 |  |  |  |  |  |  |  |  |  |  |  |  |  | 15 |
| AT/AT |  |  | 3 |  | 2 |  |  | 1 | 1 |  | 1 |  |  |  |  |  | 8 |
| CG/CG |  |  | 2 |  |  |  |  |  |  |  |  |  |  |  |  |  | 2 |
| AAC/GTT |  | 3 |  |  |  |  |  |  |  |  |  |  |  |  |  |  | 3 |
| AAG/CTT |  | 19 |  | 3 |  |  |  |  |  |  |  |  |  |  |  |  | 20 |
| AAT/ATT |  | 7 |  | 1 |  |  |  |  |  |  |  |  |  |  |  |  | 6 |
| ACT/AGT |  | 3 |  |  |  |  |  |  |  |  |  |  |  |  |  |  | 3 |
| AGC/GCT |  | 4 |  |  |  |  |  |  |  |  |  |  |  |  |  |  | 4 |
| AGG/CCT |  | 2 |  |  |  |  |  |  |  |  |  |  |  |  |  |  | 2 |
| ATC/GAT |  | 2 |  |  |  |  |  |  |  |  |  |  |  |  |  |  | 2 |
| AAAC/GTTT | 1 |  |  |  |  |  |  |  |  |  |  |  |  |  |  |  | 1 |
| AAAG/CTTT | 2 |  |  |  |  |  |  |  |  |  |  |  |  |  |  |  | 2 |
| AAAT/ATTT | 4 | 2 |  |  | 1 |  |  |  |  |  |  |  |  |  |  |  | 7 |
| AACG/GTTC | 2 |  |  |  |  |  |  |  |  |  |  |  |  |  |  |  | 2 |
| AAGG/CCTT | 2 |  |  |  |  |  |  |  |  |  |  |  |  |  |  |  | 2 |
| AAGT/ACTT | 1 |  |  |  |  |  |  |  |  |  |  |  |  |  |  |  | 1 |
| AATC/GATT | 9 |  |  |  |  |  |  |  |  |  |  |  |  |  |  |  | 9 |
| AATG/TTAC | 2 |  |  |  |  |  |  |  |  |  |  |  |  |  |  |  | 2 |
| AATT/TTAA | 1 |  |  |  |  |  |  |  |  |  |  |  |  |  |  |  | 1 |
| AGAT/TCTA |  | 7 |  | 3 |  |  |  |  |  |  |  |  |  |  |  |  | 10 |
| AGCT/TCGA | 1 |  |  |  |  |  |  |  |  |  |  |  |  |  |  |  | 1 |
| AAAGC/TTTCG | 2 |  |  |  |  |  |  |  |  |  |  |  |  |  |  |  | 2 |
| AAAGG/TTTCC | 3 | 1 |  |  |  |  |  |  |  |  |  |  |  |  |  |  | 4 |
| AAATC/TTTAG | 2 |  |  |  |  |  |  |  |  |  |  |  |  |  |  |  | 2 |
| AAATT/TTTAA | 5 |  |  |  |  |  |  |  |  |  |  |  |  |  |  |  | 5 |
| AATCG/TTAGC | 3 |  |  |  |  |  |  |  |  |  |  |  |  |  |  |  | 3 |
| AACGG/TTGCC | 1 |  |  |  |  |  |  |  |  |  |  |  |  |  |  |  | 1 |
| AAGGG/TTCCC | 2 |  |  |  |  |  |  |  |  |  |  |  |  |  |  |  | 2 |
| AATTC/TTAAG | 3 |  |  |  |  |  |  |  |  |  |  |  |  |  |  |  | 3 |
| ACCAG/TGGTC | 1 |  |  |  |  |  |  |  |  |  |  |  |  |  |  |  | 1 |
| AGAGG/TCTCC | 1 |  |  |  |  |  |  |  |  |  |  |  |  |  |  |  | 1 |
| AGGGG/TCCCC | 3 |  |  |  |  |  |  |  |  |  |  |  |  |  |  |  | 3 |
| ATATC/TATAG | 1 |  |  |  |  |  |  |  |  |  |  |  |  |  |  |  | 1 |
| AAAAAG/TTTTC | 4 |  |  |  |  |  |  |  |  |  |  |  |  |  |  |  | 4 |
| AAAAAT/TTTTA | 4 |  |  |  |  |  |  |  |  |  |  |  |  |  |  |  | 4 |
| AAACAT/TTTGTA | 1 |  |  |  |  |  |  |  |  |  |  |  |  |  |  |  | 1 |
| AAATAG/TTTATC | 5 |  |  |  |  |  |  |  |  |  |  |  |  |  |  |  | 5 |
| AAATAT/TTTATA | 3 |  |  |  |  |  |  |  |  |  |  |  |  |  |  |  | 3 |
| AAATTG/TTTAAC | 1 |  |  |  |  |  |  |  |  |  |  |  |  |  |  |  | 1 |
| AACCAG/TTGGTC | 1 |  |  |  |  |  |  |  |  |  |  |  |  |  |  |  | 1 |
| AAGCAG/TTCGTC | 3 |  |  |  |  |  |  |  |  |  |  |  |  |  |  |  | 3 |
| AAGCTC/TTCGAG | 1 |  |  |  |  |  |  |  |  |  |  |  |  |  |  |  | 1 |
| AAGCTG/TTCGAC | 1 |  |  |  |  |  |  |  |  | 2 |  |  |  |  |  |  | 3 |
| AAGGAG/TTCCTC | 2 |  |  |  |  |  |  |  |  |  |  |  |  |  |  |  | 2 |
| AATAGC/TTATCG | 2 |  |  |  |  |  |  |  |  |  |  |  |  |  |  |  | 2 |
| AATATC/TTATAG | 1 |  |  |  |  |  |  |  |  |  |  |  |  |  |  |  | 1 |
| AATGGC/TTACCG | 1 |  |  |  |  |  |  |  |  |  |  |  |  |  |  |  | 1 |
| ACACCT/TGTGGA | 1 |  |  |  |  |  |  |  |  |  |  |  |  |  |  |  | 1 |
| ACTCGC/TGAGCG | 1 |  |  |  |  |  |  |  |  |  |  |  |  |  |  |  | 1 |
| AGATCC/TCTAGG | 1 |  |  |  |  |  |  |  |  |  |  |  |  |  |  |  | 1 |
| AGCCGC/TCGGCG | 1 |  |  |  |  |  |  |  |  |  |  |  |  |  |  |  | 1 |
| AGGGGG/TCCCCC | 3 |  |  |  |  |  |  |  |  |  |  |  |  |  |  |  | 3 |
| AAATAAT/TTTATTA |  | 3 |  |  |  |  |  |  |  |  |  |  |  |  |  |  | 3 |
| AAGTGAC/TTCACTG | 1 |  |  |  |  |  |  |  |  |  |  |  |  |  |  |  | 1 |
| AATACGC/TTATGCG | 1 |  |  |  |  |  |  |  |  |  |  |  |  |  |  |  | 1 |
| AATCTGG/TTAGACC | 1 |  |  |  |  |  |  |  |  |  |  |  |  |  |  |  | 1 |
| AATGGAT/TTACCTA | 1 |  |  |  |  |  |  |  |  |  |  |  |  |  |  |  | 1 |
| ACTCCCT/TGAGGGA | 1 |  |  |  |  |  |  |  |  |  |  |  |  |  |  |  | 1 |
| AAATAATT/TTTATTAA | 1 |  |  |  |  |  |  |  |  |  |  |  |  |  |  |  | 1 |
| AAATATAT/TTTATATA | 1 |  |  |  |  |  |  |  |  |  |  |  |  |  |  |  | 1 |
| AAATTATT/TTTAATAA | 1 |  |  |  |  |  |  |  |  |  |  |  |  |  |  |  | 1 |
| AACTTACG/TTGAATGC | 1 |  |  |  |  |  |  |  |  |  |  |  |  |  |  |  | 1 |
| AACTTAATT/TTGAATTAA | 2 |  |  |  |  |  |  |  |  |  |  |  |  |  |  |  | 2 |
| AAGCAAGTG/TTCGTTCAC | 2 |  |  |  |  |  |  |  |  |  |  |  |  |  |  |  | 2 |
| ACACTATCG/TGTGATAGC |  |  |  |  |  |  | 2 |  |  |  |  |  |  |  |  |  | 2 |
| AACAGGCTAT/TTGTCCGATA | 1 |  |  |  |  |  |  |  |  |  |  |  |  |  |  |  | 1 |
| AAGATAATAGTG/TTCTATTATCAC |  |  |  | 2 |  |  |  |  |  |  |  |  |  |  |  |  | 2 |
| AAAATTGATATAT/TTTTAACTATATA | 1 |  |  |  |  |  |  |  |  |  |  |  |  |  |  |  | 1 |
| AAAACACTAGTAAT/TTTTGTGATCATTA |  |  |  | 1 |  |  |  |  |  |  |  |  |  |  |  |  | 1 |
| AAAAATGGTGAAACATG/TTTTTACCACTTTGTAC | 1 |  |  |  |  |  |  |  |  |  |  |  |  |  |  |  | 1 |
| AAAAAGGATTCAATAATCTTGCCC/ TTTTTCCTAAGTTATTAGAACGGG | 1 |  |  |  |  |  |  |  |  |  |  |  |  |  |  |  | 1 |
| Total | | | | | | | | | | | | | | | | | 386 |

**Table S3.** Distribution of hexa-, 7, 8, 9, 10, 12, 13, 14, 17 and 24-nucleotide repeats single sequence repeats (SSRs) in *Cyrtomium falcatum* chloroplast genome.

| **SSR Type** | **SSR Sequence** | **SSR Size (bp)** | **Start** | **End** | **Location** |
| --- | --- | --- | --- | --- | --- |
| hexa | (AAAAAG)×2 | 12 | 7180 | 7191 | *psbK*/*psbI* (IGS) |
| hexa | (AAAAAG)×2 | 12 | 36,303 | 36,314 | *rps14* (CDS) |
| hexa | (AAAAAG)×2 | 12 | 48,406 | 48,417 | *ndhC*/*trnV*-UAC (IGS) |
| hexa | (AAAAAG)×2 | 12 | 116,831 | 116,842 | *ndhA* (intron) |
| hexa | (AAAAAT)×2 | 12 | 2540 | 2551 | *matK* (CDS) |
| hexa | (AAAAAT)×2 | 12 | 46,491 | 46,502 | *trnF*-GAA/*ndhJ* (IGS) |
| hexa | (AAACAT)×2 | 12 | 31,470 | 31,481 | *trnS*-UGA/*psbC* (IGS) |
| hexa | (AAATAG)×2 | 12 | 4499 | 4510 | *rps16* (intron) |
| hexa | (AAATAG)×2 | 12 | 65,408 | 65,419 | *clpP* (intron) |
| hexa | (AAATAG)×2 | 12 | 78,301 | 78,312 | *rpl16* (intron) |
| hexa | (AAATAG)×2 | 12 | 93,246 | 93,257 | *rrn16*/*rps12* (IGS) |
| hexa | (AAATAT)×2 | 12 | 20,507 | 20,518 | *rpoC2*/*rpoC1* (IGS) |
| hexa | (AAATAT)×2 | 12 | 95,527 | 95,538 | *rps12* (intron) |
| hexa | (AAATAT)×2 | 12 | 125,103 | 125,114 | *ycf1* (CDS) |
| hexa | (AAATTG)×2 | 12 | 14,099 | 14,110 | *atpH*/*atpI* (IGS) |
| hexa | (AACCAG)×2 | 12 | 41,022 | 41,033 | *psaA* (CDS) |
| hexa | (AAGCAG)×2 | 12 | 13,470 | 13,481 | *atpH* (CDS) |
| hexa | (AAGCAG)×2 | 12 | 57,872 | 57,883 | *cemA* (CDS) |
| hexa | (AAGCAG)×2 | 12 | 59,180 | 59,191 | *petA* (CDS) |
| hexa | (AAGCTC)×2 | 12 | 31,987 | 31,998 | *psbC* (CDS) |
| hexa | (AAGCTG)×2 | 12 | 636 | 647 | *ndhB* (CDS) |
| hexa | (AAGGAG)×2 | 12 | 36,314 | 36,325 | *rps14* (CDS) |
| hexa | (AAGGAG)×2 | 12 | 62,883 | 62,894 | *trnP*-UGG*/psaJ* (IGS) |
| hexa | (AATAGC)×2 | 12 | 3702 | 3713 | *rps16* (CDS) |
| hexa | (AATATC)×2 | 12 | 34,907 | 34,918 | *psbD*/*trnT*-GGU (IGS) |
| hexa | (AATGGC)×2 | 12 | 33,922 | 33,933 | *psbD* (CDS) |
| hexa | (ACACCT)×2 | 12 | 34,006 | 34,017 | *psbD*/*trnT*-GGU (IGS) |
| hexa | (ACTCGC)×2 | 12 | 23,852 | 23,863 | *rpoB* (CDS) |
| hexa | (AGATCC)×2 | 12 | 1024 | 1035 | *ndhB* (CDS) |
| hexa | (AGCCGC)×2 | 12 | 31,571 | 31,582 | *psbC* (CDS) |
| hexa | (AGGGGG)×2 | 12 | 7191 | 7202 | *psbK*/*psbI* (IGS) |
| hexa | (AGGGGG)×2 | 12 | 34,218 | 34,230 | *psbD*/*trnT*-GGU (IGS) |
| hexa | (AGGGGG)×2 | 12 | 56,784 | 56,795 | *ycf4*/*cemA* (IGS) |
| 7-nucleotide | (AAATAAT)×2 | 14 | 29,355 | 29,368 | *petN*/*trnC*-GCA (IGS) |
| 7-nucleotide | (AAATAAT)×2 | 14 | 69,853 | 69,866 | *psbB*/*psbT* (IGS) |
| 7-nucleotide | (AAATAAT)×2 | 14 | 117,562 | 117,575 | *ndhA* (Intron) |
| 7-nucleotide | (AAGTGAC)×2 | 14 | 43,440 | 43,453 | *ycf3*/*trnS*-GGA (IGS) |
| 7-nucleotide | (AATACGC)×2 | 14 | 35,434 | 35,447 | *trnT*-GGU/*trnfM*-CAU (IGS) |
| 7-nucleotide | (AATCTGG)×2 | 14 | 11,429 | 11,442 | *atpA* (CDS) |
| 7-nucleotide | (AATGGAT)×2 | 14 | 34,626 | 34,639 | *psbD*/*trnT*-GGU (IGS) |
| 7-nucleotide | (ACTCCCT)×2 | 14 | 34,102 | 34,115 | *psbD*/*trnT*-GGU (IGS) |
| 8-nucleotide | (AAATAATT)×2 | 16 | 31,498 | 31,513 | *trnS*-UGA/*psbC* (IGS) |
| 8-nucleotide | (AAATATAT)×2 | 16 | 20,495 | 20,510 | *rpoC2*/*rpoC1* (IGS) |
| 8-nucleotide | (AAATTATT)×2 | 16 | 69,880 | 69,895 | *psbB*/*psbT* (IGS) |
| 8-nucleotide | (AACTTACG)×2 | 16 | 31,277 | 31,292 | *trnS*-UGA/*psbC* (IGS) |
| 9-nucleotide | (AACTTAATT)×2 | 18 | 12,911 | 12,928 | *atpF* (CDS) |
| 9-nucleotide | (AAGCAAGTG)×2 | 18 | 89,771 | 89,788 | *trnA*-UGC/*trnI*-GAU (CDS) |
| 9-nucleotide | (ACACTATCG)×7 | 63 | 105,007 | 105,069 | *ycf2*/*trnN*-GUU (IGS) |
| 10-nucleotide | (AACAGGCTAT)×2 | 20 | 29,620 | 29,639 | *petN*/*trnC*-GCA (IGS) |
| 12-nucleotide | (AAGATAATAGTG)×3 | 36 | 104,208 | 104,243 | *ycf2* (CDS) |
| 13-nucleotide | (AAAATTGATATAT)×2 | 26 | 60,234 | 60,259 | *petA*/*psbJ* (IGS) |
| 14-nucleotide | (AAAACACTAGTAAT)×5 | 70 | 45,588 | 45,657 | *trnL*-UAA (Intron) |
| 17-nucleotide | (AAAAATGGTGAAACATG)×2 | 34 | 35,733 | 35,766 | *trnT*-GGU/*trnfM*-CAU (IGS) |
| 24-nucleotide | (AAAAAGGATTCAATAATCTTGCCC)×2 | 48 | 8179 | 8226 | *trnS*-GCU/*ycf12* (IGS) |
